# Structure and Variability of the North Equatorial Current/Undercurrent from Mooring Measurements at 130°E in the Western Pacific

**DOI:** 10.1038/srep46310

**Published:** 2017-04-19

**Authors:** Linlin Zhang, Fu Jun Wang, Qingye Wang, Shijian Hu, Fan Wang, Dunxin Hu

**Affiliations:** 1 Key Laboratory of Ocean Circulation and Waves, Institute of Oceanology, Chinese Academy of Sciences, Qingdao 266071, China; 2 Laboratory for Ocean and Climate Dynamics, Qingdao National Laboratory for Marine Science and Technology, Qingdao 266237, China

## Abstract

The mean structure and variability of the North Equatorial Current/Undercurrent (NEC/NEUC) are investigated with one-year Acoustic Doppler Current Profilers measurements from 4 subsurface moorings deployed at 10.5°N, 13°N, 15.5°N, and 18°N along 130°E in the western Pacific. The strong westward flowing NEC ranges from the sea surface down to 400 m, and the mean zonal velocity of the NEC at 10.5°N is around −30 cm/s at the depth of 70 m. Eastward flowing NEUC jets are detected below the NEC at 10.5°N and 13°N, and the depth of the NEUC could reach at least 900 m. The mean velocity of the NEUC is around 4.2 cm/s at 800 m. No eastward undercurrents are observed at 15°N and 18°N. The mooring measurements also reveal a strong intraseasonal variability of the currents at all 4 mooring sites, and the period is around 70–120 days. The vertical structure of this intraseasonal variability varies at different latitudes. The variability of the NEUC jets at 10.5°N and 13°N appears to be dominated by subthermocline signals, while the variability of the currents at 15.5°N and 18°N is dominated by surface-intensified signals.

One of the dominant patterns of the tropical western Pacific ocean circulation in the upper layer is the bifurcation of the North Equatorial Current (NEC), where the westward flowing NEC impinges upon the Philippine coast and bifurcates into the northward flow-Kuroshio and the southward flow-Mindanao Current (MC)^1^,^2^. Over the past two decades, many previous studies have investigated the structure and multi-scale variability of the upper layer circulation in this area based on *in situ* observations, satellite altimetry and model outputs^3^. In comparison, our knowledge of the circulation pattern below the thermocline still remains fragmentary due to the lack of *in situ* observations.

Limited Conductivity-Temperature-Depth (CTD) transects during the TOGA/WOCE period suggest that there seems to be three countercurrents beneath the upper layer wind-driven circulation, and they are the Luzon Undercurrent below the Kuroshio, the Mindanao Undercurrent below the MC and the North Equatorial Undercurrent below the NEC, even though their structures vary in different cruises^4^,^5^,^6^,^7^. Into the 21st century, the development of Argo floats technology provides us with an effective means to investigate the subthermocline ocean circulation^8^. Recent studies detected three eastward NEUC jets below the NEC using Argo data, and these NEUC jets are zonally coherent across the whole basin from the western boundary to the eastern Pacific^9^. Shipboard ADCP measurements also show an eastward subthermocline flow which seems to be the NEUC^10^,^11^. Climatological features of the three subthermocline countercurrents and their relationships are investigated with Argo profiles combining with CTD data^12^, and results show that the northern and southern parts of the NEUC are fed by the LUC and MUC, respectively. Further investigation suggests that the subthermocline circulation in the tropical western Pacific is comprised of two components^13^. On scales shorter than 400 km, there are multiple pairs of convergent boundary currents induced by the NEUC jets. On scales longer than 400 km, there is a mean northward MUC below the MC, but no LUC is observed below the Kuroshio^13^. They further suggest that the MUC and NEUC jets are formed by nonlinear interactions of meso-scale eddies^13^,^14^. In addition, eddy-mean flow interaction and air-sea interaction mechanisms are also proposed to interpret the subthermocline zonal jets in the Pacific^15^,^16^,^17^.

In fact, the direct way to observe time series of the velocity profile of the currents is the mooring Acoustic Doppler Current Profilers (ADCP) measurements. As an important component of *in-situ* observations of the Northwestern Pacific Ocean Circulation and Climate Experiment (NPOCE) program, two subsurface moorings were deployed east of Philippine at 8°N and 18°N in December 2010 to measure the western boundary undercurrents. Two-year time series of ADCP measurements confirmed the mean northward MUC below the MC and the southward LUC below the Kuroshio, and also revealed strong intraseasonal to interannual variability of these undercurrents^18^,^19^,^20^,^21^. In comparison to the MUC and LUC, so far the existence of the NEUC jets has not been confidently confirmed by time series measurements of velocity, and their variability also remains unexplored, even though the time-mean zonal jet structures below the NEC appear in geostrophic calculations and different numerical models^4^,^12^,^14^,^22^.

During September 2014-September 2015, five subsurface ADCP moorings were deployed in the western Pacific along 130°E between 8.5°N and 18°N, and one-year time series of velocity profiles from the sea surface to 850 m were obtained. Based on these velocity records, we investigated the mean structure and variability of the NEC/NEUC in this work. The time-mean zonal NEUC jets were observationally confirmed with time series of velocity measurements, and their intraseasonal variability was revealed for the first time.

## Data and Method

**Figure 1:**
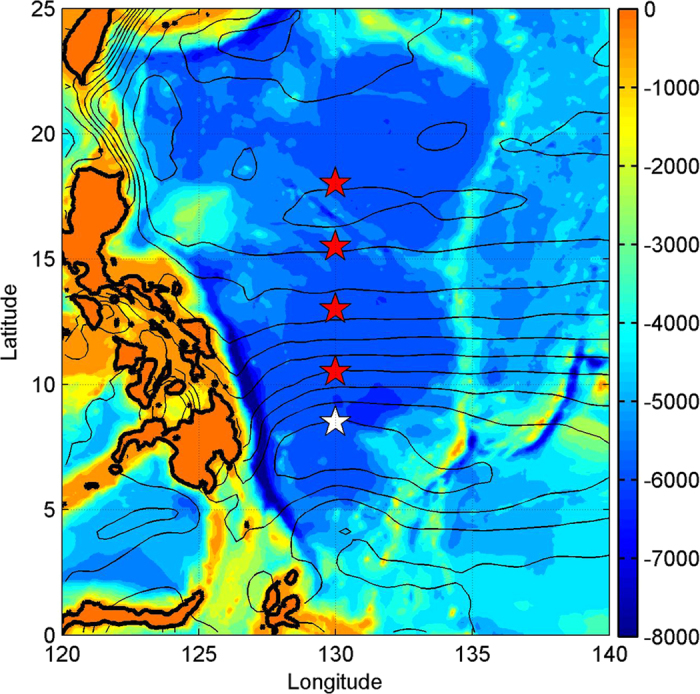
Location of the five subsurface moorings east of Philippine (star). Color shows the bathymetry of the western Pacific Ocean, and the bathymetry data is downloaded from https://www.ngdc.noaa.gov/mgg/global/. Contours show the mean sea surface height from satellite altimetry (AVISO, http://www.aviso.altimetry.fr/en/data.html). Figures are plotted using MATLAB R2010b (http://www.mathworks.com/).

Five subsurface moorings were deployed along 130°E in the western Pacific at 8.5°N, 10.5°N, 13°N, 15.5°N, and 18°N to measure the NEC/NEUC, and the water depth is around 5,500 m (Fig. 1). These mooring sites were selected generally corresponding to the three cores of the NEUC, according to previous geostrophic calculations from CTD sections and Argo floats measurements^7^,^9^.

These subsurface moorings were deployed in September 2014 and retrieved in September 2015. Unfortunately, the mooring at 8.5°N was lost due to some technical reasons, and only four moorings were retrieved successfully. For each mooring, two Acoustic Doppler Current Profilers (75-kHz) were equipped on the main float at the depth of 400 m, looking upward and downward respectively. The ADCPs were configured to measure velocities hourly with a standard bin size of 8 m, and two ADCPs were able to measure the velocity profile in the upper 850 m. The measured hourly velocity data were first processed with standard quality control procedures, and then were interpolated onto depth levels between 0 and 900 m with 10 m intervals. After that the data were daily averaged to remove the tidal signal and then used in the following analysis.

## Results

### Mean velocity structure of the NEC/NEUC

**Figure 2:**
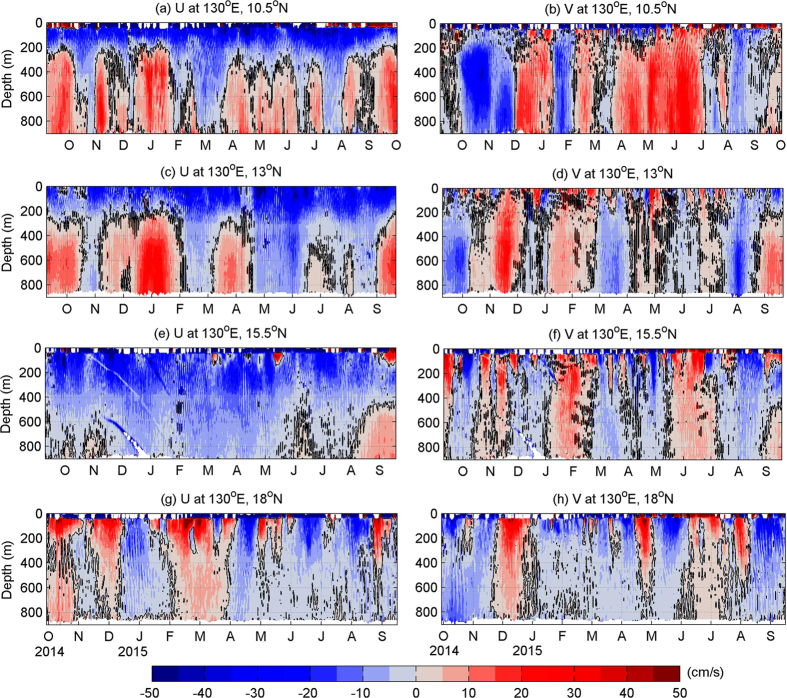
Daily mean zonal (left) and meridional (right) velocities measured by mooring ADCPs at 10.5°N (**a**,**b**), 13°N (**c**,**d**), 15.5°N (**e**,**f**), and 18°N (**g**,**h**) from September 2014 to September 2015. Black contour indicates the zero velocity line. Figures are plotted using MATLAB R2010b (http://www.mathworks.com/).

Figure 2 shows the one-year time series of the daily averaged velocities from mooring ADCPs at different latitudes. Owing to the surface wave-induced backscattering of acoustic sounds, most of the ADCPs have data blanks near the sea surface, and 50 m is probably the shallowest depth of reliable ADCP measurements in this region. Because the ADCP at 13°N well captured the structure and variability of the NEC and NEUC, we will describe the ADCP measurements at this site as an example. As shown in Fig. 2c, there is a strong and stable westward-flowing NEC in the upper 300 m at 13°N during the whole observation period. The strongest NEC appears in April 2015, and the velocity reaches −85 cm/s at the depth of 50 m. Below the depth of 300 m, there appears to be an intermittent eastward-flowing jet, and this jet seems to be strongly associated with intraseasonal events. The zonal velocity of this jet sometimes reaches up to 28 cm/s, and sometimes reduces to −14 cm/s due to those intraseasonal events. Similarly, the meridional velocity is also dominated by strong intraseasonal signals. Details of the mean structure and intraseasonal variability of this jet will be discussed in following sections.

**Figure 3:**
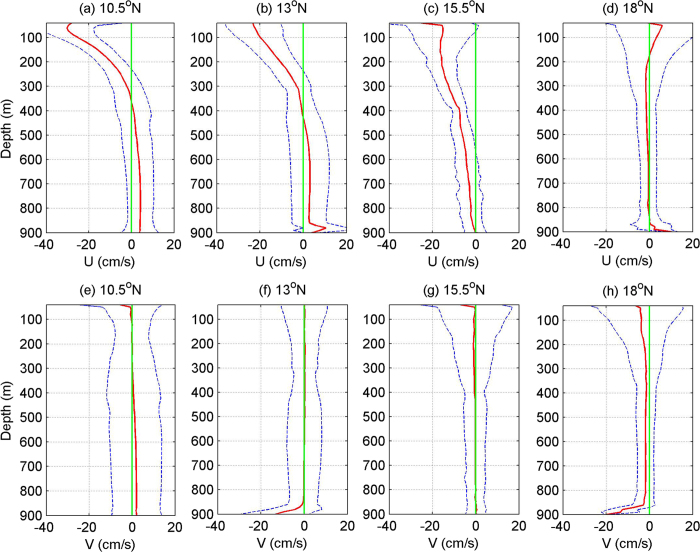
Mean zonal (upper) and meridional (lower) velocities (red) measured by mooring ADCPs at 10.5°N (**a**,**e**), 13°N (**b**,**f**), 15.5°N (**c**,**g**), and 18°N (**d**,**h**). Blue curve shows the standard deviation of the velocity, and green line indicates the zero velocity line. Figures are plotted using MATLAB R2010b (http://www.mathworks.com/).

Figure 3 shows the one-year mean zonal and meridional velocities derived from mooring ADCPs at 10.5°N, 13°N, 15.5°N and 18°N. Since the mean current in this area is dominated by the zonal component, and the mean meridional current is nearly zero (Fig. 3e,f), we focus on the zonal current in this study. The NEC is characterized by a strong westward flow in the upper 300 m, which appears at latitudes of 10.5°N, 13°N and 15.5°N. The maximum mean zonal velocity of the NEC at 10.5°N reaches −30 cm/s at about 70 m, and the standard deviation is about 12 cm/s, indicating that NEC is a very stable current (Fig. 3a). The NEC appears to weaken northward, and the maximum mean zonal velocity reduces to −23.5 cm/s and −16.5 cm/s at 13°N and 15.5°N, respectively. The westward-flowing NEC is invisible at 18°N, and the mean current at this latitude flows eastward above the depth of 200 m, and its maximum velocity is about 6 cm/s at 50 m depth (Fig. 3d), which is believed to be associated with the Subtropical Countercurrent (STCC). The STCC shows very large variances with a standard deviation of 21.6 cm/s, probably being caused by strong eddy activities in that area.

Eastward subthermocline countercurrents below the NEC are observed at 10.5°N and 13°N (Fig. 3a,b), ranging from about 400 m to 850 m–the deepest level reached by the ADCPs on our moorings. These eastward subthermocline currents are associated with the North Equatorial Undercurrent (NEUC) jets reported by previous studies^4^,^7^,^9^. The maximum mean zonal velocity of the NEUC observed by the ADCP at 10.5°N is around 4.2 cm/s at the depth of 800 m with a standard deviation of 6.2 cm/s (Fig. 3a). The NEUC at 13°N is relatively weaker, and its maximum mean zonal velocity is only 3.1 cm/s at 700 m with a standard deviation of 8.8 cm/s. Statistical significance test shows that the weak eastward mean current is below the 95% confidence level, implying that only one-year ADCP measurements are not long enough to give a robust mean current. In addition, no eastward subthermocline countercurrent are observed at 15.5°N, and the westward-flowing NEC extends from the sea surface down to 900 m at this latitude (Fig. 3c). At 18°N, the vertical profile of the mean zonal velocity shows a very weak westward flow below the STCC in the depth range of 200–500 m. Below 500 m, the mean zonal velocity at 18°N is nearly zero, indicating that there is no stable zonal currents at this depth (Fig. 3d).

The mean zonal currents crossing the 130°–135°E section in the northwestern Pacific were calculated with Argo data^9^, and our mooring ADCP measurements are generally consistent with the Argo results in terms of the mean velocity and depth range of the NEC/NEUC, confirming the existence of the NEUC jets and the effectiveness of the Argo data in resolving the mean structure of oceanic currents, even subthermocline currents. Nevertheless, the ADCP-measured mean velocity of the NEC is a little stronger than the geostrophic calculation results from Argo data, and the position of the south NEUC jet derived from Argo data seems to deviate from the ADCP-measured jet by 1°–2°, which is probably caused by the optimal interpolation error of Argo float data or different observation periods between the mooring data and Argo measurements. We also compared the mean velocity profile with another recent Argo results^12^, and they generally agree with each other. One significant difference is at 13°N, our results show countercurrents below 400 m, but Argo results show no countercurrents at this latitude. The difference might also be due to the optimal interpolation error of Argo float data, or different periods. Considering the jet-like structures of subthermocline currents, 13°N is near the boundary of the jets, therefore meridional shifts of the jets may also produce such differences.

### Intraseasonal Variability (ISV) of the NEC/NEUC

Mooring ADCP measurements not only provide the mean velocity structure of the NEC/NEUC, but also enable us to investigate the variability of these currents for the first time. As shown by the velocity time series measured by the mooring ADCP at 130°E, 13°N, the NEC/NEUC exhibits strong intraseasonal variations (Fig. 2c,d). Similarly, the currents measured by the ADCP at other latitudes also exhibit significant intraseasonal variability.

**Figure 4:**
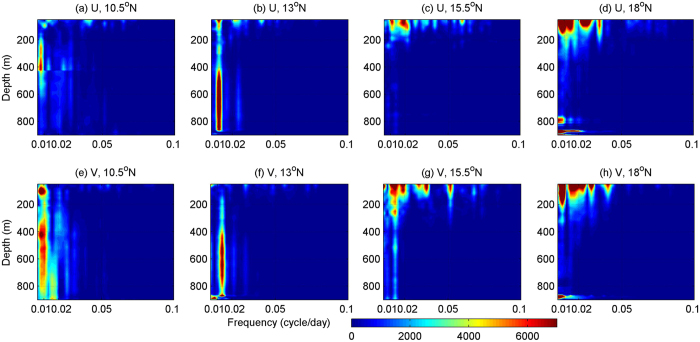
Power Spectral Density of the zonal (upper) and meridional (lower) velocity time series at different depths from mooring ADCPs at 10.5°N (**a**,**e**), 13°N (**b**,**f**), 15.5°N (**c**,**g**), and 18°N (**d**,**h**). Figures are plotted using MATLAB R2010b (http://www.mathworks.com/).

To investigate these ISV signals, we calculated the Power Spectral Density (PSD) of the zonal and meridional velocity time series at different depths using the mooring ADCP measurements (Fig. 4). The PSD of velocity at 10.5°N and 13°N generally exhibits similar features, and a protruding one is the coherent peak with the period of 70–120 days for almost the whole water column from the sea surface down to 900 m. This ISV signal is strong below the thermocline, and weakens upward. Statistical significance test shows that the ISV signal below the thermocline is significant at the 95% confidence level, but the signal above the thermocline is insignificant due to its weak strength and influences from other higher frequency fluctuations. Higher frequency fluctuations with period of 30–50 days also exits below the thermocline, but they are below the 95% confidence level. At 15.5°N and 18°N, the PSD distribution is significantly different from that at 10.5°N and 13°N. The dominant feature of the PSD is multiple surface-intensified peaks with period ranging from 15 to 120 days. The strongest peak corresponds to the period of 70–120 days. This signal weakens downward, and is able to reach the depth of 900 m. Peak periods of other higher-frequency fluctuations range from 15 to 50 days, varying at different latitudes. Differing from the 70–120 days ISV, these higher frequency fluctuations only exist in the upper 400 m. Statistical significance test shows that the ISV with period of 70–120 days is significant only below 200 m, and ISV with period of 15–50 days is significant only above 200 m. Recent mooring observations east of Philippine also show strong ISVs of the western boundary currents but with slightly different peak period at different locations^18^,^19^,^23^. The connection of ISVs of these different currents in the western Pacific and their dynamics needs further investigations.

To better understand the ISV signal, we filtered the daily velocity time series using a 10–180 days band-pass Butterworth filter, and the filtered velocities clearly show the vertically coherent ISV signal of the NEC/NEUC between 0–850 m (not shown). Strong ISV signals appear below the depth of 400 m at 10.5°N and 13°N, and the amplitude of these ISV signals reaches 20 cm/s. In contrast, ISV signals above 400 m are relatively weak, implying that ISV signals at these two latitudes are probably caused by subthermocline processes. At 15.5°N and 18°N, the ISV signal seems different, which is strong in the surface and weakens with depth, indicating influences from traditional surface-intensified meso-scale processes.

**Figure 5:**
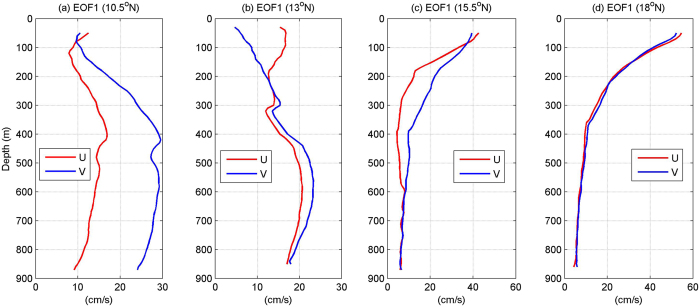
First EOF mode of the zonal (red) and meridional (blue) velocity time series measured by mooring ADCPs at 10.5°N (**a**), 13°N (**b**), 15.5°N (**c**), and 18°N (**d**). Figures are plotted using MATLAB R2010b (http://www.mathworks.com/).

**Figure 6:**
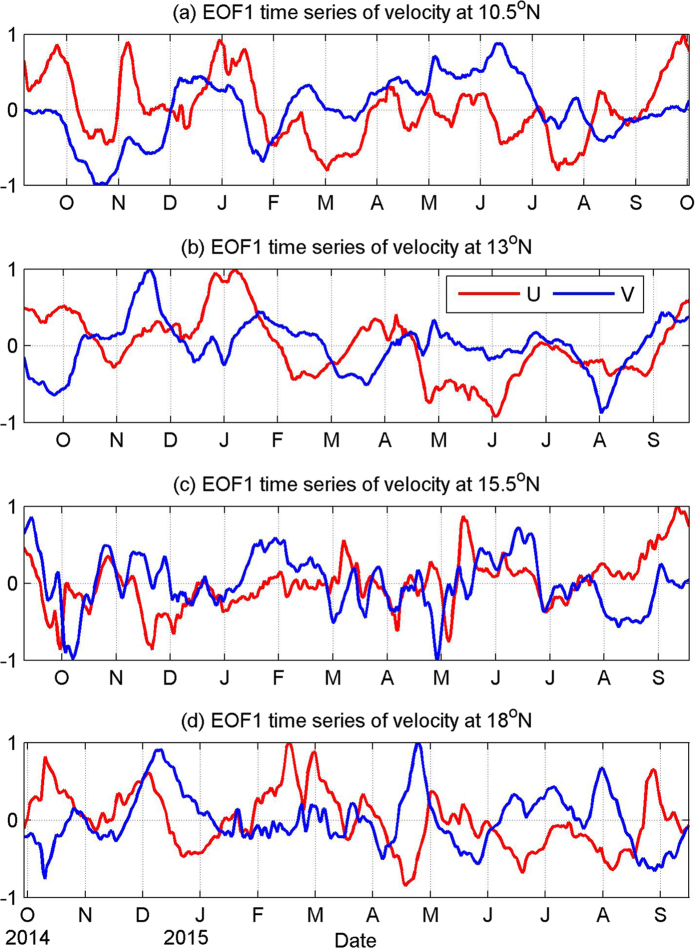
Time series of the first EOF mode of the zonal (red) and meridional (blue) velocity measured by mooring ADCPs at 10.5°N (**a**), 13°N (**b**), 15.5°N (**c**), and 18°N (**d**). Figures are plotted using MATLAB R2010b (http://www.mathworks.com/).

**Figure 7:**
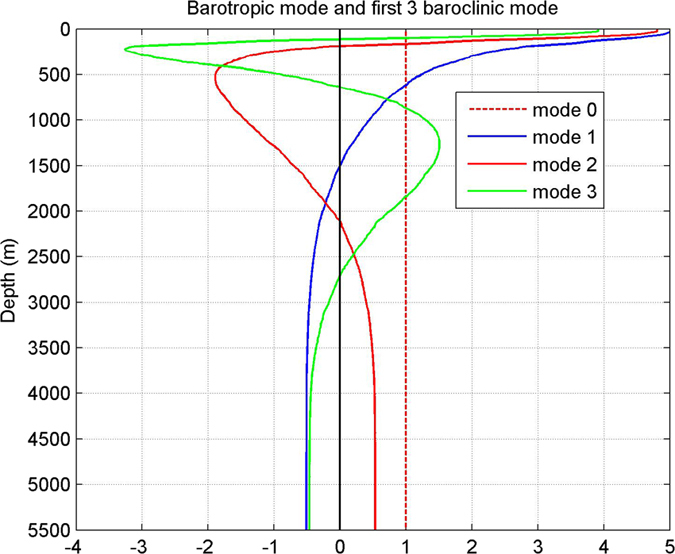
Barotroic mode and first three baroclinic mode profiles calculated with full-depth CTD data in the NEC/NEUC area. Figures are plotted using MATLAB R2010b (http://www.mathworks.com/).

To investigate the vertical structure of these ISVs statistically, we calculated the empirical orthogonal function (EOF) mode of the velocity time series measured by the mooring ADCP. The pattern and associated time coefficient of the first EOF mode are shown in Figs 5 and 6, separately. For both zonal and meridional velocities at all four mooring sites, the first EOF mode captures most part of the total velocity variance. For zonal velocity, EOF1 explains 67%, 80%, 58% and 86% of the total variance at 10.5°N, 13°N, 15.5°N and 18°N, respectively. For meridional velocity, the percentage is 88%, 77%, 73% and 85%, respectively. The statistical significance of the first EOF mode has been verified based on the method proposed by previous studies^24^. As shown in Fig. 5c,d, the first EOF mode of both zonal and meridional velocities at 15.5°N and 18°N seems to have similar structures. They are obviously surface intensified, and generally resemble the first baroclinic mode profile calculated with the stratification data in this area based on vertical standing mode decomposition^25^ (Fig. 7). Time series of the first EOF mode also exhibits significant intraseasonal signals (Fig. 6c,d), implying that ISV signals at these latitudes is closely associated with the first baroclinic mode and mainly reflects the variation of the thermocline. Differently, at 10.5°N and 13°N where the NEUC jets appear, the first EOF mode of the currents seems intensified below the thermocline with the strongest signals appearing between 400–700 m (Fig. 5a,b). This vertical structure is totally different from the first baroclinic mode, but seems to be related to the second baroclinic mode which has a similar subsurface intensified feature below the thermocline (Fig. 7). However, the first EOF mode does not show surface-intensified features above 200 m as the second baroclinic mode does, implying that ISV signals of the NEUC jets at these two latitudes are probably also affected by other baroclinic modes.

**Figure 8:**
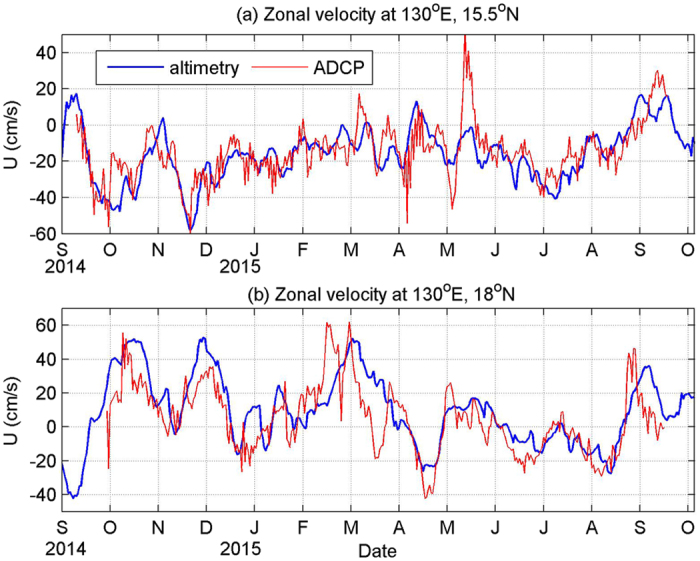
Daily zonal geostrophic velocity derived from satellite altimetry (blue) and daily mean zonal velocity at 70 m measured by mooring ADCPs (red) at 15.5°N (**a**) and 18°N (**b**). Satellite altimetry data is downloaded from http://www.aviso.altimetry.fr/en/data.html. Figures are plotted using MATLAB R2010b (http://www.mathworks.com/).

Strong surface-intensified ISV signals at 15.5°N and 18°N make it possible for satellite altimeters to capture these signals. We compared zonal geostrophic velocities derived from satellite altimetry with velocity measurements at 70 m from mooring ADCP (Fig. 8). Time series measured by two independent instruments appear to coincide well with each other. At 15.5°N, the ADCP-measured velocity leads the altimetry results by 1 day with a correlation of 0.7 and root-mean square (RMS) of 12 cm/s. At 18°N, it leads the altimetric velocity by 3 days with a correlation of 0.75 and RMS of 15 cm/s. These high correlations enable us to use satellite altimetry to investigate the ISV of the currents. Satellite altimetry demonstrates that there are very strong eddy activities in this region owing to the baroclinic instability of the currents^26^, and ISV signals observed by mooring ADCPs at 15.5°N and 18°N are primarily caused by these meso-scale eddies. Obviously, ISVs at these four mooring sites can be categorized into surface-intensified mode and subthermocline mode. Even though surface-intensified ISVs at 15.5°N and 18°N have no significant correlations, they are supposed to be governed by similar mechanisms. As mentioned above, meso-scale eddies caused by baroclinic instability of the currents are the source of these ISVs. For the subthermocline ISVs at 10.5°N and 13°N, they are significantly correlated with each other, and the correlation is 0.36. These subthermocline ISVs are believed to be associated with subthermocline eddies in this area.

Based on model outputs and a 1.5 layer nonlinear reduced gravity model, westward propagating mesoscale eddies caused by the breakdown of the annual mode-1 baroclinic Rossby waves are suggested to form the time-mean NEUC jets^14^, implying that the ISV of the NEUC jets is caused by those mesoscale eddies with first baroclinic mode structures. However, the mooring measurements in this study revealed that the vertical structures of those ISVs vary in different regions, and the subthermocline mode seems to dominate the variability of the currents at 10.5°N and 13°N where the NEUC jets appear. Results from an eddy-resolving ocean general circulation model also suggest that there are westward propagating subthermocline eddies^27^ generated by interactions between meridional shifts of the NEC, NEUC and their interactions with the topography east of Philippine, and the propagation speed is much slower than the first mode baroclinic Rossby wave. In fact, the large standard deviation of the vertical mode of the velocity variations in the upper 600 m implies that the vertical mode of the variations is different in different regions, even though its basin-wide average is in the first baroclinic mode^14^ (see their Fig. 4a), agreeing with our results. Generally speaking, it seems that westward propagating subthermocline eddies may play an important role in the intraseasonal variability of the NEUC jets, and the mechanism for the generation of these subthermocline eddies will be investigated in future studies.

## Conclusion and Discussion

Based on one-year time series of ADCP measurements from four subsurface moorings deployed at 130°E in the northwestern Pacific, the mean velocity structure and variability of the NEC/NEUC were investigated in this study. The existence of eastward flowing NEUC jets below the NEC are confirmed for the first time with time series of current measurements at 10.5°N and 13°N, though its mean velocity is relatively weak. The mooring ADCP measurements also revealed strong intraseasonal variability of the NEC/NEUC with a period of 70–120 days at all four mooring sites. The vertical structure of the intraseasonal variability varies in different regions, and the subthermocline mode dominates the variability at latitudes where the NEUC jets appear.

Several studies based on theoretical analysis or model simulations have indicated that zonal jets in the Pacific are possibly caused by nonlinear interactions of geostrophic turbulence on the beta-plane^22^,^28^,^29^. The NEUC jets are suggested to originate from annual baroclinic Rossby waves caused by the large-scale wind forcing over the eastern Pacific^14^. Mesoscale eddies caused by the breakdown of the annual baroclinic Rossby waves propagate westward and form time-mean zonal jets by the converging potential vorticity fluxes. Based on altimeter measurements, an eddy-mean flow interaction mechanism is also proposed^15^, in which the presence of the mean zonal jets in the ocean regularizes the formation of mesoscale eddies which in return feed back to maintain the jets. In the South Pacific, there are also subthermocline zonal jets below the South Equatorial Current, and the collocated, small-scale wind forcing seems to contribute to the formation of these jets^16^. According to coupled model results^17^, the subthermocline zonal jets in the South Pacific could generate fine-scale SST anomalies through the zonal advection process in the temperature gradient area, and induce fine-scale wind stress curl anomalies that reinforce the subthermocline jets again through the Sverdrup dynamics. However, the role of such air-sea interaction mechanism in generating the subthermocline zonal jets (e.g., NEUC) in the North Pacific still remains unexplored. As mentioned in the last section, subthermocline eddy activities are very active in this area, which may also play an important role in the formation of the mean jets through the ‘turbulent Sverdrup balance’^28^, therefore it is important for future studies to investigate the interaction between the NEUC jets and the subthermocline eddies. In addition, it should be noted that this study is only based on mooring measurements at 130°E, it is also necessary for future studies to examine the variability of the NEUC jets at other longitudes with more observations or model outputs.

## Additional Information

**How to cite this article**: Zhang, L. *et al*. Structure and Variability of the North Equatorial Current/Undercurrent from Mooring Measurements at 130°E in the Western Pacific. *Sci. Rep.* **7**, 46310; doi: 10.1038/srep46310 (2017).

**Publisher's note:** Springer Nature remains neutral with regard to jurisdictional claims in published maps and institutional affiliations.

## References

[b1] Fine R., Lukas R., Bingham F., Warner M. & Gammon R. The western equatorial Pacific: A water mass crossroads. J. Geophys. Res. 99(C12), 25,063–25,080 (1994).

[b2] Lukas R., Yamagata T. & McCreary J. Pacific low-latitude western boundary currents and the Indonesian throughflow. J. Geophys. Res. 101(C5), 12,209–12,216 (1996).

[b3] Hu D. . Pacific western boundary currents and their roles in climate. Nature 522, 299–308 (2015). 26085269 10.1038/nature14504

[b4] Hu D. & Cui M. The western boundary current of the Pacific and its role in the climate. Chin. J. Oceanol. Limnol. 9, 1–14 (1991).

[b5] Qu T., Kagimoto T. & Yamagata T. A subsurface countercurrent along the east coast of Luzon. Deep Sea Res. 44, 413–423 (1997).

[b6] Qu T., Mitsudera H. & Yamagata T. On the western boundary currents in the Philippine Sea. J. Geophys. Res. 103, 7537–7548 (1998).

[b7] Wang F., Hu D. & Bai H. Western boundary undercurrents east of the Philippines. *Proceedings of the 4th Pacific Ocean Remote Sensing Conference (PORSEC)* 551–556 (1998).

[b8] Roemmich D. . The Argo program: Observing the global ocean with profiling floats. Oceanography 22, 34–43 (2009).

[b9] Qiu B., Rudnick D., Chen S. & Kashino Y. Quasistationary North Equatorial Undercurrent jets across the tropical North Pacific Ocean. Geophys. Res. Lett. 40, 2183–2187 (2013).

[b10] Kashino Y. . Observations of the North Equatorial Current, Mindanao Current, and Kuroshio Current System during the 2006/07 El Niño and 2007/08 La Niña. J. Oceanogr. 65, 325–333 (2009).

[b11] Dutrieux P. Tropical western Pacific currents and the origin of intraseasonal variability below the thermocline. Ph.D. thesis, University of Hawaii at Manoa, pp.122 (2009).

[b12] Wang F., Zang N., Li Y. & Hu D. On the subsurface countercurrents in the Philippine Sea. J. Geophys. Res. Oceans 120, 131–144 (2015).

[b13] Qiu B., Chen S., Rudnick D. & Kashino Y. A new paradigm for the North Pacific subthermocline low-latitude western boundary current system. J. Phys. Oceanogr. 45, 2407–2423 (2015).

[b14] Qiu B., Chen S. & Sasaki H. Generation of the North Equatorial Undercurrent jets by triad baroclinic Rossby wave interactions. J. Phys. Oceanogr. 43, 2682–2698 (2013).

[b15] Maximenko N., Bang B. & Sasaki H. Observational evidence of alternating zonal jets in the world ocean. Geophys. Res. Lett. 32, L12607, doi: 10.1029/2005GL022728 (2005).

[b16] Kessler W. & Gourdeau L. Wind-driven zonal jets in the South Pacific Ocean. Geophys. Res. Lett. 33, L03608, doi: 10.1029/2005GL025084 (2006).

[b17] Taguchi B. . Deep oceanic zonal jets constrained by fine-scale wind stress curls in the South Pacific Ocean: A high-resolution coupled GCM study. Geophys. Res. Lett. 39, L08602, doi: 10.1029/2012GL051248 (2012).

[b18] Hu D. . Direct measurements of the Luzon Undercurrent. J. Phys. Oceanogr. 43, 1417–1425 (2013).

[b19] Zhang L. . Mindanao Current/Undercurrent measured by a subsurface mooring. J. Geophys. Res. Oceans 119, 3617–3628 (2014).

[b20] Chen Z. . Strengthening Kuroshio observed at its origin during November 2010 to October 2012. J. Geophys. Res. Oceans 120, doi: 10.1002/2014JC010590 (2015).

[b21] Hu S. . Interannual variability of the Mindanao Current/Undercurrent in direct observations and numerical simulations. J. Phys. Oceanogr. 46, 483–99 (2016).

[b22] Nakano H. & Hasumi H. A series of zonal jets embedded in the broad zonal flows in the Pacific obtained in eddy-permitting ocean general circulation models. J. Phys. Oceanogr. 35, 474–488 (2005).

[b23] Kashino Y., Ishida A. & Kuroda Y. Variability of the Mindanao Current: Mooring observation results. Geophys. Res. Lett. 32, L18611, doi: 10.1029/2005GL023880 (2005).

[b24] North G., Bell T., Cahalan R. & Moeng F. Sampling Errors in the Estimation of Empirical Orthogonal Functions. Mon. Wea. Rev. 110, 699–706 (1982).

[b25] Philander G. El & Nino La Nina, and the Southern Oscillation. 293pp (Academic Press, 1990).

[b26] Qiu B. Seasonal eddy field modulation of the North Pacific Subtropical Countercurrent: TOPEX/Poseidon observations and theory. J. Phys. Oceanogr. 29, 2471–2486 (1999).

[b27] Chiang T., Wu C., Qu T. & Hsin Y. Activities of 50–80 day subthermocline eddies near the Philippine coast. J. Geophys. Res. Oceans 120, 3606–3623 (2015).

[b28] Rhines P. & Holland W. A theoretical discussion of eddy-driven mean flows, Dyn. Atmos. Oceans 3, 289–325 (1979).

[b29] Richards K., Maximenko N., Bryan F. & Sasaki H. Zonal jets in the Pacific Ocean. Geophys. Res. Lett. 33, L03605, doi: 10.1029/2005GL024645 (2006).

